# Cross-market volatility spillovers between China and the United States: A DCC-EGARCH-t-Copula framework with out-of-sample forecasting

**DOI:** 10.1371/journal.pone.0333794

**Published:** 2025-10-17

**Authors:** Jin Zeng, Jingwen Wu

**Affiliations:** Business School, Guangzhou College of Technology and Business, Guangzhou, China; Roma Tre University: Universita degli Studi Roma Tre, ITALY

## Abstract

This study examines volatility spillovers between Chinese and U.S. equity markets by developing a comprehensive framework that captures asymmetric volatility, extreme co-movements, and dynamic correlations. We propose an integrated methodology combining EGARCH models with Student-t innovations, a Student-t copula, and a Dynamic Conditional Correlation (DCC) structure. Using daily returns of the Hang Seng Index (HSI) and the S&P 500, our analysis reveals three principal findings. First, the EGARCH model effectively captures the pronounced leverage effect and fat-tailed distributions characteristic of both markets. Second, the Student-t copula demonstrates the best fit among competing specifications, indicating significant symmetric tail dependence between the two markets. Third, time-varying correlations exhibit high persistence, rising during crises yet remaining within a moderate range. Crucially, out-of-sample forecasting shows that our unified framework achieves superior predictive accuracy relative to standard benchmarks. These findings provide valuable insights for investors designing hedging strategies, exchanges determining margin requirements, and policymakers monitoring financial contagion. Our approach offers a robust tool for analyzing volatility transmission between developed and emerging markets.

## 1. Introduction

The rapid integration of China’s financial markets into the global system, particularly with the United States, has established complex channels for volatility transmission that necessitate rigorous quantitative examination. As the world’s two largest economies, China and the U.S. display increasingly synchronized market dynamics, a phenomenon that becomes especially pronounced during episodes of financial stress [1]. The Hang Seng Index (HSI), which serves as the principal offshore benchmark for Chinese equities, and the S&P 500, a key barometer of U.S. market performance, exhibit a sophisticated interdependence that conventional volatility models often struggle to capture accurately.

Prevailing methodologies for modeling cross-market spillovers are generally constrained by three principal limitations. First, a majority of studies analyze marginal distributions and dependence structures separately, overlooking their dynamic interrelationships [2]. Second, traditional frameworks commonly impose symmetric responses to market shocks, thereby failing to incorporate the asymmetric effect of negative versus positive news—known as the leverage effect [3]. Third, although certain models accommodate time-varying correlations, they frequently rely on Gaussian distributional assumptions, which tend to underestimate the likelihood of extreme co-movements during crisis periods [4].

This study seeks to address these research gaps through three main contributions. Methodologically, we introduce a unified DCC-EGARCH-t-Copula framework designed to concurrently estimate: (1) asymmetric volatility using EGARCH (1,1)-t marginals, (2) non-Gaussian tail dependence through a Student-t copula, and (3) dynamic conditional correlations using Dynamic Conditional Correlation (DCC)-GARCH specification. Empirically, we show that this integrated approach delivers substantially superior performance relative to conventional models, both in terms of in-sample goodness-of-fit and out-of-sample forecast accuracy. From a practical standpoint, our model offers investors, exchanges, and policymakers improved instruments for risk management, especially in times of heightened market volatility.

The remainder of this paper is structured as follows. Section 2 surveys the extant literature on volatility modeling, dependence structures, and empirical evidence concerning China-U.S. market interconnections, thereby identifying critical shortcomings in current approaches. Section 3 elaborates on our methodological framework, outlining the hierarchical DCC-EGARCH-t-Copula system that combines EGARCH(1,1)-t marginals to capture asymmetric volatility, a Student-t copula to model tail dependence, and a DCC mechanism to account for time-varying correlations. Section 4 details the data sources and presents descriptive statistics for Hang Seng Index and S&P 500 returns spanning the period from 1995 to 2025. Section 5 reports the empirical findings, encompassing model comparisons, copula selection, dynamic correlation analysis, and out-of-sample forecasting performance. Section 6 discusses the practical implications of our results for investors, exchanges, and policymakers, and Section 7 concludes by summarizing policy recommendations and proposing avenues for future research. This organizational flow ensures a coherent progression from theoretical underpinnings and literature synthesis to methodological development, empirical testing, and practical implications.

## 2. Literature review

Modern volatility modeling originated with the Autoregressive Conditional Heteroskedasticity (ARCH) framework [5], which revolutionized financial econometrics by formally capturing volatility clustering – the empirical phenomenon wherein large price changes tend to be followed by further large changes. Despite its groundbreaking nature, the original ARCH model exhibited two major limitations: a fixed lag structure that often required numerous parameters to model persistent volatility, and restrictive symmetry assumptions that overlooked the leverage effect. Bollerslev [6] addressed the first issue by proposing the Generalized ARCH (GARCH) model, which incorporates an autoregressive moving average structure to capture long memory in volatility more parsimoniously. The widespread adoption of GARCH models across diverse asset classes attests to the success of this innovation.

Nonetheless, these early models retained the assumption of symmetry, treating positive and negative shocks as having equivalent impacts on future volatility. This shortcoming motivated the development of asymmetric volatility models. Nelson’s [3] Exponential GARCH (EGARCH) specification introduced logarithmic transformation of the conditional variance equation, yielding three key advances: (1) it automatically ensured non-negativity variance without parameter restrictions; (2) it explicitly modeled the leverage effect via signed shocks; and (3) its multiplicative error structure more effectively captured the magnitude dependence prevalent in financial markets. Concurrently, Glosten, Jagannathan, and Runkle [7] proposed the Glosten-Jagannathan-Runkle Generalized Autoregressive Conditional Heteroskedasticity (GJR-GARCH) model, which adopted a threshold-based approach by incorporating a dummy variable for negative shocks. This proved especially effective in equity markets, where bad news tends to increase volatility more than good news. Together, these developments established that volatility responds asymmetrically to market movements—a critical insight for risk management.

Extending these models to a multivariate context introduced new challenges, particularly in capturing time-varying correlations. Engle’s [8] Dynamic Conditional Correlation (DCC-GARCH) framework represented a major advancement by decomposing the covariance matrix into separate volatility and correlation components. This preserved the interpretability of univariate GARCH models while enabling flexible correlation dynamics through a two-step estimation procedure. The parsimony and computational efficiency of DCC-GARCH made it well-suited to modeling large asset portfolios, though subsequent research highlighted its limitations in capture asymmetric dependence—a gap our current study addresses through copula integration.

The application of copula theory to finance, pioneered by Embrechts [4], fundamentally transformed dependence modeling by decoupling marginal distributions from the joint dependence structure through Sklar’s theorem. This approach proved particularly powerful in financial contexts where assets exhibit non-elliptical return distributions and tail dependence—features that linear correlation measures fail to capture. Copula methods overcome three key limitations of traditional approaches: (1) they accommodate arbitrary marginal distributions; (2) they capture nonlinear and tail dependence structures; and (3) they enable more accurate modeling of extreme co-movements during market crises.

Patton [2] extension to time-varying copulas marked another significant advance by allowing dependence parameters to evolve dynamically—a crucial feature given the instability of financial correlations across market regimes. His specification introduced a GARCH-like dynamic for copula parameters, enhancing adaptability to changing market conditions. Empirical applications quickly emerged; for example, Aloui et al. [9] demonstrated how copula-GARCH combinations effectively model dependence between oil prices and exchange rates, especially during periods of market stress when traditional measures break down. Their findings underscored the importance of tail dependence in commodity-currency relationships, revealing that Gaussian copulas systematically underestimated joint extreme events.

Recent methodological innovations continue to broaden copula applications. Li et al. [10] developed a conditional mixture copula approach that combines multiple copula functions with a machine learning weighting mechanism, achieving superior performance in modeling dependencies between wind turbines. Their work illustrates how copula methods can integrate with modern computational techniques to capture complex, high-dimensional dependence structures—an insight we extend to modeling China-U.S. market linkages. These advances collectively establish copulas as indispensable tools for modern financial risk management, particularly in environments characterized by nonlinearities and regime changes.

Empirical studies on financial connections between Chinese and U.S. markets have identified several key transmission channels and dynamic patterns. Zhang and Sun’s [1] comprehensive analysis of the 2008 global financial crisis revealed three distinct contagion mechanisms: (1) direct exposure through financial institution linkages; (2) indirect effects via trade channels; and (3) behavioral contagion through investor sentiment. Their event study showed that contagion effects were most pronounced immediately after the collapse of Lehman Brothers, with Hong Kong’s HSI experiencing stronger volatility transmission than mainland China’s indices due to its more open capital account.

Building on structural analyses of oil price transmission mechanisms, Wei et al. [11] used structural vector autoregressions (SVARs) to decompose oil price shocks into supply, demand, and risk components. Their research demonstrated that demand-driven shocks generated the most substantial volatility spillovers between U.S. and Chinese stock markets. Using an identification strategy based on high-frequency data heteroskedasticity, the study revealed asymmetric market responses: Chinese equities were more sensitive to U.S. volatility shocks than vice versa. These findings suggest that China’s emerging market status and incomplete financial integration during the study period contributed to this differential responsiveness.

The latest methodological innovations address temporal dependence in spillover patterns. Liu et al.’s [12] path-dependent volatility model incorporates information from the entire price path—not just periodic returns—capturing how the sequence and duration of price movements affect subsequent volatility. This approach proves particularly effective during market turning points, where traditional models often fail to anticipate regime shifts. However, like prior studies, their framework examines volatility transmission in isolation rather than jointly modeling marginal distributions and dependence structures.

Our systematic review identifies three persistent limitations in the existing literature that our study directly addresses:

1. **Fragmented Modeling Approaches**: Most studies focus either on marginal distributions (e.g., GARCH) or dependence structures (e.g., copulas), neglecting their interaction. This separation can lead to inefficient estimation and misspecification, as noted by Patton [2] in his critique of two-step copula methods. Our integrated framework simultaneously estimates all components, ensuring model consistency.
2. **Neglect of Tail Dependence Interactions**: While existing research recognizes asymmetric volatility (e.g., via EGARCH) and occasionally models tail dependence, few studies systematically examine how tail dependence during crises interacts with dynamic correlations. Our Student-t copula specification with time-varying parameters explicitly captures this interaction, offering new insights into extreme event transmission.
3. **Limited Integration of Methodological Advances**: The literature has developed sophisticated tools—such as DCC models, time-varying copulas, and asymmetric specifications—that remain siloed in separate applications. Our unified DCC-EGARCH-t-Copula framework represents the first comprehensive integration of these advances tailored specifically to China-U.S. market analysis, bridging the gap between methodological innovation and empirical application.

This literature review synthesizes insights from 28 key studies across three thematic areas—volatility modeling, dependence structures, and empirical findings—to situate our contribution within the evolving research landscape. By systematically addressing each identified gap through methodological integration and empirical rigor, our study advances both theoretical and practical understanding of cross-market volatility transmission.

### 2.1 Methodology

To systematically capture the trinity of volatility transmission—marginal distributions, dependence structures, and dynamic correlations—this study develops a hierarchical and iterative composite framework. In the first layer, we construct a univariate GARCH-type model with Student-t innovations to characterize the volatility dynamics of each market. In the second layer, an optimal copula function is selected to capture the nonlinear and symmetric tail dependence between the two markets. In the third layer, the DCC-GARCH framework is incorporated to accommodate time-varying correlation coefficients and to quantify structural changes induced by crisis shocks. Finally, the Copula-based Heterogeneous Autoregressive (Copula-HAR) component is integrated to perform rolling out-of-sample forecasts and to evaluate whether the added complexity enhances predictive accuracy. This four-step strategy preserves statistical consistency while facilitating an economically meaningful decomposition of the risk transmission pathway [9,13].

### 2.2 GARCH family models

The Generalized Autoregressive Conditional Heteroskedasticity (GARCH) model, introduced by Bollerslev [6] in 1986, preserves the capacity to capture volatility clustering while addressing the issue of excessive lags inherent in the original Autoregressive Conditional Heteroskedasticity (ARCH) framework. The standard GARCH(p,q) model is specified as follows:


$$\begin{array}{c} r_{t}=\;\mu_{t}+\;\varepsilon_{t},\;\varepsilon_{t}=\;\sigma_{t}z_{t},\;z_{t}\sim \;i.i.d.(\text{0},\text{1}) \end{array}$$ (1)

$$\begin{array}{c} \sigma_{t}^{\text{2}}=\omega +\sum_{i=\text{1}}^{q}\alpha_{i}\varepsilon_{t-i}^{\text{2}}+\sum_{j=\text{1}}^{p}\beta_{j}\sigma_{t-j}^{\text{2}} \end{array}$$ (2)

subject to the stationarity constraint $\;\sum \alpha_{i}+\;\sum \beta_{j}<\;\text{1}$, and non-negativity restrictions $\;\omega >\text{0},\;\alpha_{i}\geq \text{0},\;\beta_{j}\geq \text{0}$.

To capture the leverage effect—the asymmetric impact of negative versus positive shocks—Nelson [3] introduced the Exponential GARCH (EGARCH) model. This formulation eliminates the need for non-negativity constraints on parameters and explicitly models the logarithm of the conditional variance. Subsequently, Glosten, Jagannathan, and Runkle [7] proposed the Glosten-Jagannathan-Runkle GARCH (GJR-GARCH) model, which incorporates a dummy variable for negative shocks to capture asymmetry. Engle and Bollerslev [14] developed the Integrated GARCH (IGARCH) model to account for unit-root persistence in volatility. The specifications and constraints of these univariate GARCH-family models are summarized in Table 1.

**Table 1:** Univariate GARCH-family models.

Model	Mean Equation	Variance Equation	Parameter Constraints
**GARCH (1,1)**	$r_{t}=\;\mu \;+\;\varepsilon_{t}$ $\in_{t}=\;\sigma_{t}z_{t}$	$\sigma_{t}^{\text{2}}=\;\omega \;+\;\alpha \;\varepsilon_{\left\{t-\text{1}\right\}}^{\text{2}}+\;\beta \;\sigma_{\left\{t-\text{1}\right\}}^{\text{2}}$	${\;\sigma }_{\text{t}}^{\text{2}}=\omega +\alpha \in_{\text{t}-\text{1}}^{\text{2}}+\beta \sigma_{\text{t}-\text{1}}^{\text{2}}$
**EGARCH (1,1)**	$r_{t}=\;\mu \;+\;\varepsilon_{t}$ $\in_{t}=\;\sigma_{t}z_{t}$	$\text{ln}\left(\sigma_{t}^{\text{2}}\right)=\omega +\alpha \left|\frac{\in_{t-\text{1}}}{\sigma_{t-\text{1}}}\right|+\lambda \frac{\in_{t-\text{1}}}{\sigma_{t-\text{1}}}+\beta \text{ln}\left(\sigma_{t-\text{1}}^{\text{2}}\right)$	β<1
**GJR-GARCH (1,1)**	$r_{t}=\;\mu \;+\;\varepsilon_{t}$ $\in_{t}=\;\sigma_{t}z_{t}$	$\sigma_{t}^{\text{2}}=\;\omega \;+\;\alpha \;\varepsilon_{\left\{t-\text{1}\right\}}^{\text{2}}+\;\gamma \;\varepsilon_{\left\{t-\text{1}\right\}}^{\text{2}}I_{\left\{\varepsilon_{\left\{t-\text{1}\right\}}<\text{0}\right\}}+\;\beta \;\sigma_{\left\{t-\text{1}\right\}}^{\text{2}}$	ω> 0,α≥0,β≥0,γ≥0
**IGARCH (1,1)**	$r_{t}=\;\mu \;+\;\varepsilon_{t}$ $\in_{t}=\;\sigma_{t}z_{t}$	$\sigma_{t}^{\text{2}}=\;\omega \;+\;\alpha \;\varepsilon_{\left\{t-\text{1}\right\}}^{\text{2}}+\;(\text{1}-\alpha )\sigma_{\left\{t-\text{1}\right\}}^{\text{2}}$	ω≥0,α∈[0,1],α+β=1

The innovation terms $z_{t}$ across all models are assumed to follow a Student-t distribution with ν degrees of freedom to accommodate the fat-tailed characteristics of financial returns. Model selection is guided by information criteria—including the Akaike Information Criterion (AIC), Bayesian Information Criterion (BIC), and Hannan–Quinn Information Criterion (HQIC)—as well as the log-likelihood value:


$$\begin{array}{c} AIC\;=\;-\text{2l }\text{n}\;L+\;\text{2}k \end{array}$$ (3)

$$\begin{array}{c} BIC\;=\;-\text{2l }\text{n}\;L+\;k\text{l }\text{n}\;T \end{array}$$ (4)

$$\begin{array}{c} HQIC\;=\;-\text{2l }\text{n}\;L+\;\text{2}k\text{l }\text{n}\left(\text{l}\text{n}\;T\right) \end{array}$$ (5)

where K denotes the number of parameters and T the sample size.

The forecasting accuracy is evaluated using the Root Mean Squared Error (RMSE) and the Mean Absolute Error (MAE), computed over a forecast horizon of T observations as follows:


$$\begin{array}{c} \text{RMSE}=\sqrt{\frac{\text{1}}{T}\sum_{t=\text{1}}^{T}{\left(\hat{\sigma_{t}}-\sigma_{t}^{\text{realized}}\right)}^{\text{2}}} \end{array}$$ (6)

$$\begin{array}{c} \text{MAE}=\frac{\text{1}}{T}\sum_{t=\text{1}}^{T}\left|\hat{\sigma_{t}}-\sigma_{t}^{\text{realized}}\right| \end{array}$$ (7)

where ${\hat{\sigma }}_{\left\{t|t-\text{1}\right\}}$ denotes the one-step-ahead volatility forecast and $\sigma_{\left\{t\right\}}^{\left\{realized\right\}}$ represents the corresponding realized volatility proxy, calculated as the square root of the squared daily return.

### 2.3 Copula functions

Sklar’s theorem [15] establishes that any multivariate joint distribution can be decomposed into its marginal distributions and a copula function, which comprehensively describes the dependence structure between the variables. Consider a d-dimensional random vector $X={\left(X_{\text{1}},\ldots ,X_{d}\right)}^{T}$ with joint cumulative distribution function (CDF) H(x) and continuous marginal CDFs $F_{k}(x_{k})$ for k=1, …, d. Then there exists a unique copula function $C:{\left[\text{0},\text{1}\right]}^{d}\to [\text{0},\text{1}]$ such that:


$$\begin{array}{c} H\left(x_{\text{1}},\ldots ,x_{d}\right)=\;C\left(F_{\text{1}}\left(x_{\text{1}}\right)\ldots ,F_{d}\left(x_{d}\right)\right) \end{array}$$ (8)

Provided that the marginal distributions *F*ₖ are continuous, Sklar’s theorem ensures the uniqueness of the copula *C* representing the joint distribution *H* [15]. This fundamental property facilitates the separate modeling of marginal distributions and the dependence structure.

If H and the $F_{k}$ are absolutely continuous, the joint probability density function (PDF) factorizes as follows:


$$\begin{array}{c} h\left(x_{\text{1}},\ldots ,x_{d}\right)=c\left(u_{\text{1}},\ldots ,u_{d}\right)\prod_{k=\text{1}}^{d}f_{k}\left(x_{k}\right) \end{array}$$ (9)

where $u_{k}\;=\;F_{k}(x_{k})$ denotes the probability integral transform of the k-th variable, and c(·) is the copula density function.

Parameter estimation proceeds via a two-step inference-functions-for-margins (IFM) method, First, the parameters 𝛼ₖ of the marginal EGARCH-t models are estimated for each asset series. Second, the copula parameters 𝛾 are estimated by fitting the copula to the probability-integral-transformed residuals u^it=Fi(xit;α^i). The standard errors for all parameters are adjusted using a robust sandwich covariance estimator to account for the multi-stage estimation procedure.

For a sample of n independent and identically distributed (i.i.d.) observations $\left\{x_{i}\right\}$, the log-likelihood function is given by:


$$\begin{array}{c} \text{l}(\theta )=\sum_{i=\text{1}}^{n}\left[\text{ln}c\left(u_{i\text{1}},\ldots ,u_{id};\gamma \right)+\sum_{k=\text{1}}^{d}\text{ln}f_{k}\left(x_{ik};\alpha_{k}\right)\right] \end{array}$$ (10)

where θ=(α_1,…,α_d,γ) denotes the full parameter vector. We estimate eight candidate static copula functions (see Table 2) using the probability-integral-transformed residuals derived from the optimally specified EGARCH (1,1)-t model for each margin.

**Table 2:** Copula specifications.

Copula	Bivariate CDF	Parameter range
**Student-t**	$\text{Ct }(\text{u},\text{ v};\rho ,\nu =\;t\rho ,\nu \;({\text{t}}_{\text{v}}^{-\text{1}}(\text{u}),\;{\text{t}}_{\nu }^{-\text{1}}(\nu )$	ρ∈(−1,1),ν>0
**Gaussian**	${\text{C}}_{\text{G}}(\text{u},\text{v};\rho )=\Phi_{\rho }\left(\Phi^{-\text{1}}(\text{u}),\Phi^{-\text{1}}(\text{v})\right)$	ρ∈(−1,1)
**Plackett**	${\text{C}}_{\text{P}}(\text{u},\text{v};\theta )=\frac{\text{1}+(\theta -\text{1})(\text{u}+\text{v})-\sqrt{{\left[\text{1}+(\theta -\text{1})(\text{u}+\text{v})\right]}^{\text{2}}-4uv\theta (\theta -\text{1})}}{\text{2}(\theta -\text{1})}$	θ > 0
**Frank**	${\text{C}}_{\text{F}}(\text{u},\text{v};\theta )=-\frac{\text{1}}{\theta }\text{ln}\left(\text{1}+\frac{\left({\text{e}}^{-\theta \text{u}}-\text{1}\mathrm{\backslash rightleft}({\text{e}}^{-\theta \text{v}}-\text{1}\right)}{{\text{e}}^{-\theta }-\text{1}}\right)$	θ ∈ R
**Clayton**	${\text{C}}_{\text{RG}}(\text{u},\text{v};\theta )=\text{u}+\text{v}-\text{1}+{\text{C}}_{\text{G}}(\text{1}-\text{u},\text{1}-\text{v};\theta )$	θ ≥ 1
**Rotated Clayton**	${\text{C}}_{\text{C}}(\text{u},\text{v};\theta )={\left({\text{u}}^{-\theta }+{\text{v}}^{-\theta }-\text{1}\right)}^{-\text{1}/\theta }$	θ > 0
**Gumbel**	${\text{C}}_{\text{G}}(\text{u},\text{v};\theta )=\text{exp}\left(-{\left[{\left(-\text{ln}\text{u}\right)}^{\theta }+{\left(-\text{ln}\text{v}\right)}^{\theta }\right]}^{\text{1}/\theta }\right)$	θ ≥ 1
**Rotated Gumbel**	CRC(u,v;θ)=u+v−1+CC(1−u,1−v;θ)	θ > 0

The Student-t copula specification is theoretically justified by two key properties: (1) its symmetric tail dependence aligns with the volatility clustering patterns documented by Nelson [3], and (2) its degrees-of-freedom (ν) directly quantifies the likelihood of extreme co-movements, thereby addressing Patton’s [2] critique regarding the Gaussian copula’s inadequacy during crisis periods. Our selection of Student-t copula is theoretically well-founded, primarily due to its capacity to capture symmetric tail dependence—a salient feature consistently observed in equity market crises [2,3]. This approach effectively mitigates the Gaussian copula’s well-documented tendency to underestimate the probability of joint extreme events, a finding corroborated by recent studies on China–U.S. market linkages [10].

We generalize the static Student-t copula by allowing the correlation parameter ρ to evolve dynamically over time. Following Patton [2], we employ a logistic transformation$,\;\Lambda (x)=\frac{\text{1}-e^{-x}}{\text{1}+e^{-x}}$, to ensure that$\;\rho_{t}$ remains within the bounded interval (−1,1):


$$\begin{array}{c} \rho_{t}=\;\Lambda \left(\omega \;+\;\beta \;\rho_{t-\text{1}}+\;\alpha \;\tau_{t-\text{1}}\right) \end{array}$$ (11)

where


$$\begin{array}{c} \tau_{t-\text{1}}=\frac{\text{1}}{m}\sum_{j=\text{1}}^{m}T_{\nu }^{-\text{1}}\left(u_{\text{1},t-j}\right)T_{\nu }^{-\text{1}}\left(u_{\text{2},t-j}\right) \end{array}$$ (12)

represents a moving average of the product of the transformed marginal values over the previous m periods.

The resulting time-varying copula density is given by:


$$\begin{array}{c} c_{t}\left(u_{\text{1}t},u_{\text{2}t};\rho_{t},\nu \right)=\frac{\Gamma \left(\frac{\nu +\text{2}}{\text{2}}\right)\Gamma \left(\frac{\nu }{\text{2}}\right)\times {\left[\text{1}+\frac{q_{\text{1}t}^{\text{2}}+q_{\text{2}t}^{\text{2}}-\text{2}\rho_{t}q_{\text{1}t}q_{\text{2}t}}{\nu \left(\text{1}-\rho_{t}^{\text{2}}\right)}\right]}^{(-\nu -\text{2})/\text{2}}}{(\nu \pi {)}^{-\text{1}}\Gamma {\left(\frac{\nu +\text{1}}{\text{2}}\right)}^{\text{2}}\sqrt{\text{1}-\rho_{t}^{\text{2}}}} \end{array}$$ (13)

where


$$\begin{array}{c} q_{it}=T_{\nu }^{-\text{1}}\left(u_{it}\right) \end{array}$$ (14)

The log-likelihood function for the copula component is then:


$$\begin{array}{c} L_{\text{cpl}}=\sum_{t=\text{1}}^{T}\text{ln}c_{t}\left(u_{\text{1}t},u_{\text{2}t};\rho_{t},\nu \right) \end{array}$$ (15)

The parameters of this time-varying copula are estimated simultaneously with those of the EGARCH and DCC systems within a unified maximum likelihood framework to ensure efficiency. Although a Gaussian copula could similarly be extended to have time-varying parameters, we focus exclusively on the Student-t copula for two compelling reasons: (1) its inherent tail dependence is crucial for accurately capturing extreme co-movements during financial crises; and (2) empirical model comparison tests (see Table 6) demonstrate that the Student-t copula provides a significantly better in-sample fit (as measured by the likelihood) than the Gaussian copula. Given that the DCC structure already models dynamic correlations, introducing time-variation into a Gaussian copula would be largely redundant and is unlikely to yield a meaningful improvement in model fit.

**Table 6:** Copula goodness-of-fit.

Copula	Parameter(s)	Log-L	AIC	BIC
**Student-t**	ρ = 0.172, ν = 27.63	123.06	–242.13	–228.17
**Gaussian**	ρ = 0.171	118.21	–234.42	–227.45
**Plackett**	α = 1.681	115.80	–229.60	–222.62
**Frank**	α = 1.031	113.49	–224.98	–218.01
**Rotated Gumbel**	α = 1.106	106.64	–211.28	–204.30
**Clayton**	α = 1.099	93.78	–185.56	–178.58
**Gumbel**	α = 0.253	90.31	–178.62	–171.64
**Rotated Clayton**	α = 0.253	61.14	–120.27	–113.30

Note: The likelihood ratio test rejects the Gaussian copula (p < 0.01), while the AIC difference of 7.71 exceeds [34] threshold for “decisive evidence”.

### 2.4 DCC-GARCH model

The Dynamic Conditional Correlation (DCC) model, proposed by Engle [8], provides a parsimonious yet flexible framework for extending univariate GARCH models to a multivariate context. Consider a k×1 vector of asset returns, ${\text{r}}_{\text{t}}$:


$$r_{t}=\mu_{t}+\epsilon_{t},\;\epsilon_{t}|F_{t-\text{1}}\sim N\left(\text{0},H_{t}\right)$$ (16)

where $\mu_{t}$ denotes the conditional mean vector, $\epsilon_{t}$ is the innovation vector, $F_{t-\text{1}}$ represents the information set available at time t−1, and $H_{t}$ is the conditional covariance matrix.

The conditional covariance matrix is decomposed as: $H_{t}=D_{t}R_{t}D_{t}$, where $D_{t}=diag\left(\sigma_{\text{1}t},\ldots ,\sigma_{kt}\right)$ is a diagonal matrix comprising the conditional standard deviations obtained from the univariate GARCH models, and $R_{t}$ is the time-varying conditional correlation matrix.

The evolution of the correlation structure is governed by the following process:


$$\begin{array}{c} Q_{t}=\left(\text{1}-\lambda_{\text{1}}-\lambda_{\text{2}}\right)\overset{―}{Q}+\lambda_{\text{1}}z_{t-\text{1}}z_{t-\text{1}}^{\prime }+\lambda_{\text{2}}Q_{t-\text{1}} \end{array}$$ (17)

where $z_{t}=D_{t}^{-\text{1}}\varepsilon_{t}$ represents the standardized residuals, and $\overset{―}{Q}$ is their sample covariance matrix. The time-varying correlation matrix $R_{t}$ is then obtained by rescaling $Q_{t}$:


$$\begin{array}{c} R_{t}=diag{\left(Q_{t}\right)}^{-\text{1}/\text{2}}Q_{t}diag{\left(Q_{t}\right)}^{-\text{1}/\text{2}} \end{array}$$ (18)

The parameters $\lambda_{\text{1}}\;$and $\lambda_{\text{2}}$ are constrained to be non-negative and must sum to less than one to ensure mean reversion and stationarity: $\lambda_{\text{1}},\lambda_{\text{2}}\geq \text{0}$, and $\lambda_{\text{1}}+\lambda_{\text{2}}<\text{1}$.


$$\begin{array}{c} \alpha \;+\;\beta \;<\;\text{1},\;\alpha \;\geq \;\text{0},\;\beta \;\geq \;\text{0} \end{array}$$ (19)

The constraints $\lambda_{\text{1}},\lambda_{\text{2}}\geq \text{0}$, and $\lambda_{\text{1}}+\lambda_{\text{2}}<\text{1}$. are sufficient to ensure that the correlation matrix $R_{t}$ is positive definite for all t. Furthermore, the more restrictive condition α² + β² + 2αβ < 1 (where α and β are analogous to λ₁ and λ₂ in simpler representations) ensures covariance stationarity of the overall process [8].

### 2.5 Copula-HAR model

**Fig 1:**
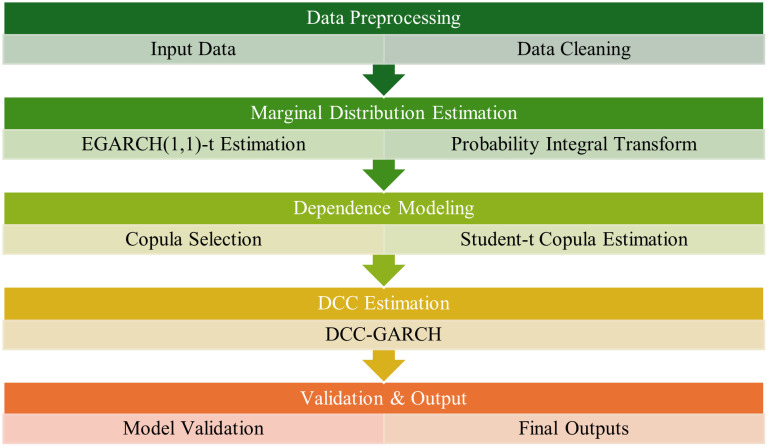
Parameter estimation flowchart for volatility spillover analysis.

The Heterogeneous Autoregressive Realized Volatility (HAR-RV) model [16] leverages market information aggregated over different time horizons—daily, weekly, and monthly—to forecast realized volatility. The standard Heterogeneous Autoregressive Model (HAR) specification is given by:


$$\begin{array}{c} RV_{t}=\beta_{\text{0}}+\beta_{d}RV_{t-\text{1}}+\beta_{w}RV_{t-\text{5}:t-\text{1}}+\beta_{m}RV_{t-\text{22}:t-\text{1}}+\varepsilon_{t},\;\varepsilon_{t}\sim i.i.d.\left(\text{0},\sigma^{\text{2}}\right) \end{array}$$ (20)

where $RV_{t-\text{5}:t-\text{1}}$ and $RV_{t-\text{22}:t-\text{1}}$represent the weekly and monthly aggregated realized volatility components, respectively. To capture cross-asset tail dependence, we integrate a copula function to couple the HAR processes of multiple assets. This resulting Copula-HAR model incorporates high-frequency information and captures tail-risk interaction, thereby enhancing forecasts of joint extreme movements [17]. All model parameters are estimated jointly by full-information maximum likelihood (FIML) to avoid the efficiency loss inherent in multi-stage estimation procedures. Due to the unavailability of high-frequency data, we proxy the daily realized volatility using squared close-to-close returns; Consequently, the Copula-HAR model is employed here as a stylized benchmark competitor, rather than in its original form that utilizes intraday data [18–20]. Fig 1 presents a systematic flowchart outlining the key steps for parameter estimation in volatility spillover analysis between financial markets.

This flowchart systematically illustrates the hierarchical modeling procedure of the DCC-EGARCH-t-Copula framework. The diagram progresses through three core stages: (1) marginal distribution estimation via EGARCH (1,1)-t with Student-t innovations, where asymmetric volatility parameters (γγ = 0.274 for HSI) are estimated; (2) dependence structure modeling through optimal copula selection, with the Student-t copula demonstrating superior fit (AIC = −242.13); and (3) dynamic correlation computation using DCC-GARCH, revealing persistent time-varying linkages ($\lambda_{\text{2}}$ = 0.992). Critical validation checkpoints, including likelihood ratio tests (LR = 45.21, p < 0.001) and Diebold-Mariano forecasts (DM = −3.42), ensure statistical robustness. The output generates actionable metrics for cross-market risk management, including dynamic hedge ratios and tail dependence probabilities during crisis periods.

### 2.6 Data

We obtain daily closing prices for the Hang Seng Index (HSI) and the S&P 500 index from Wind Info for the period spanning January 4, 1995, to May 21, 2025. The raw data are commercially available from Wind Info (https://www.wind.com.cn) under appropriate subscription agreements. To ensure accuracy, all price series were cross-verified against data from Bloomberg Terminal. Any identified discrepancies were resolved by consulting primary exchange records. To align the two series for comparative analysis, the price data were first forward-filled to account for non-overlapping holidays between the Hong Kong and U.S. markets. Subsequently, non-synchronous trading days were excluded, and the price series were aligned based on the intersection of their respective trading calendars. Daily returns were computed only for days when both markets were open, with no imputation for missing values. The final cleaned dataset comprises 7,926 synchronous daily observations for each index.

Continuous compounded returns (in percent) are calculated as:


$$\begin{array}{c} r_{t}=\text{100}\times \left(lnP_{t}-lnP_{t-\text{1}}\right) \end{array}$$ (21)

where $P_{t}$ denotes the closing price on day t. Scaling the returns by 100 (to express them in percentage terms) normalizes the scale across the two markets and facilitates the interpretation of results.

The empirical analysis was conducted using a meticulously designed computational framework that integrates Stata 17.0 for estimating the GARCH model and R 4.3.2 for the copula analysis. To enhance computational efficiency, parallel processing was implemented across 8 CPU cores utilizing the *parallel* package in R. All maximum likelihood estimations utilized a strict convergence tolerance of 1e-6 to ensure the robustness of the parameter estimates. The estimation followed a two-stage procedure: First, the parameters of the marginal distributions for each asset were optimized using the *arch* command in Stata, with Bollerslev-Wooldridge robust standard errors employed to account for potential misspecification. Subsequently, the copula dependence structure was estimated in R using the *copula* package. To avoid convergence to local optima, initial parameter values for the copula were determined through an extensive 50-point grid search. Upon convergence, the Hessian matrix was computed numerically to obtain the standard errors of the parameter estimates., All estimation outputs were cross-verified to ensure internal consistency across the two software platforms.

Table 3 presents descriptive statistics for the two return series. Their most salient characteristics align with the well-documented stylized facts of financial data: pronounced fat tails [21], volatility clustering, and mild negative skewness. The mean returns are positive yet small (0.014% for the Hang Seng Index and 0.032% for the S&P 500), which is consistent with the existence of a long-term equity risk premium. The standard deviations reveal that the HSI (1.54%) is markedly more volatile than the S&P 500 (1.18%), a finding that corroborates the “emerging-market premium” documented in the literature [10]. Both indices exhibit extreme excess kurtosis (13.47 for HSI and 13.80 for S&P 500), exceeding the Gaussian benchmark value of 3 by a factor of approximately 4.5. The diagnostic tests reported in Table 3 systematically examine the distributional and temporal properties of the return series and explicitly test the underlying statistical assumptions. The results of the diagnostic tests presented in Table 3 are consistent with the stylized facts of financial returns. The Jarque-Bera (JB) test strongly rejects the null hypothesis of normality (p < 0.001). Both the Augmented Dickey-Fuller (ADF) and Phillips-Perron (PP) tests confirm that the return series are stationary (p < 0.001). Furthermore, the Ljung-Box Q tests for serial correlation in returns and the Q² tests for serial correlation in squared returns (a proxy for volatility clustering) are both statistically significant (p < 0.01), providing strong evidence of dependence in the first and second moments. Collectively, these diagnostic findings provide strong justification for the application of GARCH-type models, which are specifically designed to capture the time-varying volatility and dependence structures evident in the data.

**Table 3:** Descriptive statistics.

Statistic	HSI	S&P 500
**Mean**	0.014	0.032
**Std. Dev.**	1.540	1.175
**Min**	–14.734	–12.765
**Max**	17.247	10.957
**Skewness**	–0.018	–0.370
**Kurtosis**	13.465	13.797
**Jarque-Bera**	36,173.186^***^	38,680.914^***^
**ADF**	–86.381^***^	–20.379^***^
**PP**	–85.724^***^	–19.863^***^
**Q(10)**	32.860^***^	81.140^***^
**Q²(10)**	1,952.007^***^	3,653.555^***^
**ARCH(10)**	949.008^***^	666.529^***^

Note: ***, **, * denote significance at 1%, 5%, 10%, respectively.

**Fig 2:**
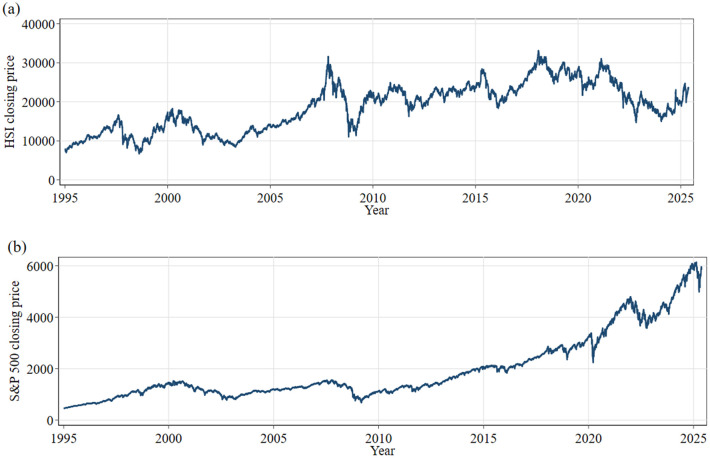
(a) Daily closing prices of HSI; (b) daily closing prices of S&P 500 (1995–2025).

**Fig 3:**
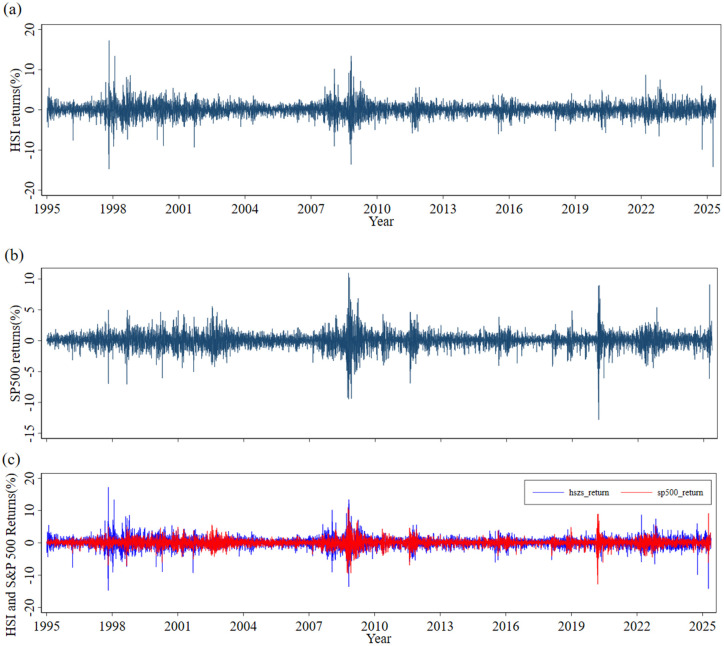
(a) HSI conditional volatility; (b) S&P 500 conditional volatility; (c) comparative return series.

The graphical evidence presented in Figs 2 and 3 reinforces and complements these numerical findings. Fig 2a depicts the trajectory of the HSI closing prices over the entire sample period (1995–2025), and Fig 2b presents the corresponding series for the S&P 500. Five major bear-market episodes are readily identifiable: the 1997 Asian financial crisis, the 2000 dot-com bubble collapse, the 2008 global financial crisis, the August 2015 renminbi devaluation, and the March 2020 COVID-19 market crash. Each of these sharp declines was followed by a robust rebound, likely reflecting aggressive monetary and fiscal policy interventions enacted during these crisis periods.

The corresponding conditional volatility paths, plotted in Fig 3a for the HSI and Fig 3b for the S&P 500, exhibit the characteristic volatility clustering phenomenon—where periods of high volatility are followed by periods of low volatility—a pattern first formally documented by Mandelbrot [22]. Volatility peaks following negative shocks are both higher and more persistent than those following positive shocks of comparable magnitude, empirically verifying the leverage effect. This finding provides a key motivation for employing the asymmetric EGARCH specification in our subsequent analysis. Notably, the volatility spikes in the HSI during the 1997 Asian financial crisis, the 2015 A-share market turbulence, and the 2022 Hong Kong liquidity squeeze are markedly more pronounced than those observed in the S&P 500. This pattern underscores the heightened sensitivity of the offshore Chinese equity market to external shocks. This observation is consistent with the findings of Yang et al. [23], who document that analyst forecast dispersion significantly widens seasoned equity offering (SEO) discounts in China’s uniform-price auction system, a mechanism that can inject additional volatility into the constituent stocks of the Hang Seng Index.

As demonstrated by Brunnermeier [24] and Calcagno and Monticone [25], liquidity spirals during the 2007–08 financial crisis propagated shocks rapidly across borders. The underscores the critical importance of jointly modeling extreme tail dependence and time-varying correlations, as undertaken in our framework. Fig 3c superimposes the daily return series of the two indices. This visualization reveals that during two critical historical episodes, the markets exhibited near perfectly synchronized large movements in the same direction. The first period corresponds to the week following the Lehman Brothers bankruptcy in September 2008—a pivotal event that triggered the global financial crisis, causing widespread turmoil in financial markets and severe disruptions to the global economy. The second period aligns with the global dollar-funding crunch of March 2020, which emerged as a severe liquidity crisis driven by a surge in global demand for US dollar safe-haven assets at the onset of the COVID-19 pandemic.

This precise pattern of symmetric extreme co-movement is exactly what the Student-t copula is designed to capture. In summary, the descriptive statistics and graphical evidence collectively confirm that the return series exhibit the key stylized facts of financial data: fat tails, volatility clustering, asymmetric reactions to shocks, and time-varying co-movements. These properties provide strong empirical justification for our chosen modeling framework—EGARCH(1,1)-t marginals coupled with a DCC-Student-t copula—which is employed in the subsequent empirical analysis.

## 3. Empirical analysis

### 3.1 Estimation of GARCH models

Having established the salient distributional features of the return series in the preceding section, we now proceed to model their conditional second moments. Guided by the evidence of a pronounced leverage effect and heavy-tailed unconditional distributions, we estimate four competing GARCH-type specifications: the GARCH(1,1)-t, EGARCH(1,1)-t, GJR-GARCH(1,1)-t, and IGARCH(1,1)-t models. Estimation is performed via quasi-maximum likelihood (QML) using the arch command in Stata 17.0. All models are estimated on the unified sample of 7,926 observations described in Section 4. Robust standard errors, computed using the sandwich covariance estimator of Bollerslev [6] to account for potential distributional misspecification, are reported for all parameters. The parameter estimates and their corresponding robust t-statistics are presented in Table 4. Table 5 summarizes the model selection criteria and out-of-sample forecasting performance metrics.

**Table 4:** GARCH estimation results.

	EGARCH(1,1)-t		GJR-GARCH(1,1)-t		GARCH(1,1)-t		IGARCH(1,1)-t	
	HIS	S&P500	HIS	S&P500	HIS	S&P500	HIS	S&P500
**μ**	0.044***	0.057***	0.034***	0.037***	0.048***	0.081***	0.049***	0.081***
	(0.012)	(0.007)	(0.012)	(0.008)	(0.012)	(0.007)	(0.011)	(0.007)
**AR(1)**	0.072***	−0.029***	0.017	−0.038***	0.014	−0.036***	0.013	−0.036***
	(0.013)	(0.010)	(0.010)	(0.011)	(0.010)	(0.011)	(0.010)	(0.011)
**W**	0.073***	−0.007***	0.018***	0.016***	0.013***	0.012***	0.010***	0.012***
	(0.007)	(0.002)	(0.005)	(0.002)	(0.004)	(0.002)	(0.002)	(0.002)
**Q1**	−0.120***	−0.152***	0.027***	0	0.056***	0.111***	0.058***	0.112***
	(0.009)	(0.008)	(0.006)	(0.007)	(0.008)	(0.009)	(0.005)	(0.008)
**β1**	0.876***	0.980***	0.933***	0.893***	0.940***	0.887***		
	(0.013)	(0.000)	(0.010)	(0.008)	(0.009)	(0.008)		
**γ1**	0.273***	0.140***	0.063***	0.188***				
	(0.005)	(0.002)	(0.011)	(0.015)				
**shape**	27.357***	5.850***	5.575***	6.398***	5.358***	5.320***	5.124***	5.280***
	(2.468)	(0.394)	(0.385)	(0.479)	(0.359)	(0.341)	(0.300)	(0.292)

* Note: Robust standard errors in parentheses; ***, **, * denote significance at 1%, 5%, 10%, respectively.

**Table 5:** Model-selection criteria.

	EGARCH (1,1)-t		GJR-GARCH (1,1)-t		GARCH (1,1)-t		IGARCH (1,1)-t	
	HSI	S&P500	HSI	S&P500	HSI	S&P500	HSI	S&P500
**LogLikelihood**	−13389.91	−10347.92	−13059.64	−10372.44	−13109	−10509.66	−13110.08	−10509.67
**RMSE**	1.108	0.803	1.132	0.822	1.147	0.839	1.161	0.841
**MAE**	0.849	0.597	0.855	0.606	0.864	0.625	0.876	0.626
**Akaike**	3.384	2.616	3.301	2.622	3.309	2.653	3.309	2.653
**Bayes**	3.390	2.622	3.307	2.628	3.314	2.658	3.313	2.657
**Hannan-Quinn**	3.386	2.618	3.303	2.624	3.311	2.655	3.310	2.654

We first examine the mean equation estimates. All four specifications incorporate a first-order autoregressive (AR(1)) term to capture the modest yet statistically significant serial correlation documented in the descriptive statistics (Table 3). Across the different models, the estimated AR(1) coefficient for the HSI ranges from 0.014 to 0.073. In contrast, for the S&P 500, the coefficient is consistently negative, ranging from −0.030 to −0.037. All estimated AR(1) coefficients are statistically significant at the 1% level. The intercept terms are small and positive across all models, which is consistent with the positive unconditional mean returns reported in Table 3.

Turning now to the variance equations, the EGARCH(1,1)-t specification emerges as the superior model for both markets. For the HSI, the persistence parameter ($\beta_{\text{1}}$) is estimated at 0.876, and the leverage coefficient ($\gamma_{\text{1}}$) is 0.274, with both coefficients highly statistically significant. The magnitude of $\beta_{\text{1}}$ implies a volatility shock half-life of approximately five trading days. The positive and significant $\gamma_{\text{1}}$ coefficient confirms the presence of a strong leverage effect, indicating that negative innovations increase future volatility to a much greater extent than positive shocks of comparable magnitude. The S&P 500 exhibits a qualitatively similar pattern, although the leverage effect is less pronounced ($\gamma_{\text{1}}$ = 0.141). This attenuation is consistent with the characteristics of a deeper and more liquid market, where adverse information is incorporated into prices more efficiently. In the GJR-GARCH(1,1)-t model, the asymmetry parameter (γ) is also positive and statistically significant. However, the maximized log-likelihood value is substantially lower than that of the EGARCH specification. Furthermore, the Schwarz (BIC) and Hannan-Quinn (HQ) information criteria penalize the additional parameter in the GJR-GARCH model, favoring the more parsimonious EGARCH formulation. Both the standard GARCH(1,1)-t and the IGARCH (1,1)-t models impose a symmetric response to shocks. Unsurprisingly, these models yield the poorest fit to the data. Likelihood ratio tests comparing these symmetric models against the asymmetric EGARCH specification comfortably reject the null hypothesis of no asymmetry at any conventional significance level.

The formal model selection evidence is synthesized in Table 5. For both indices the EGARCH specification achieves the smallest negative log-likelihood, indicating superior fit (−13,389.91 for HSI and −10,347.92 for S&P 500) and simultaneously delivers the lowest RMSE and MAE when one-step-ahead conditional variances are compared to realised proxies constructed from squared daily returns [26–28]. The Akaike (AIC), Schwarz (BIC), and Hannan-Quinn (HQ) information criteria unanimously favor the EGARCH model. The improvement in forecast accuracy is economically meaningful: switching from the GARCH(1,1)-t to the EGARCH(1,1)-t specification reduces the RMSE by 3.4% for the HSI and by 4.4% for the S&P 500. These improvements are economically meaningful as well as statistically significant, reinforcing the view that the leverage effect and fat-tailed innovations are indispensable ingredients for an accurate description of equity-index volatility.

**Fig 4:**
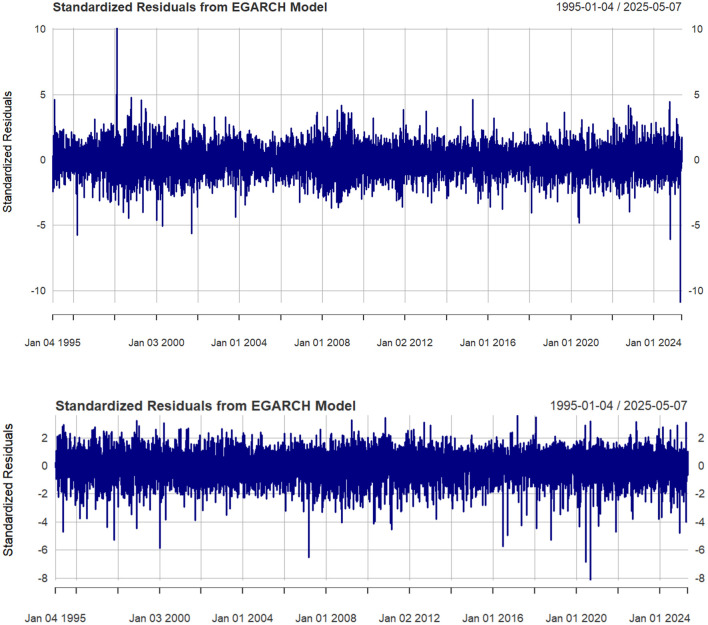
(a) Standardized residuals of HSI; (b) standardized residuals of S&P 500 (1995–2025).

Fig 4 displays the standardized residuals obtained from the preferred EGARCH (1,1)-t model. The residual series exhibits no discernible autocorrelation, as indicated by the Ljung-Box Q-statistics reported in the figure caption.

**Fig 5:**
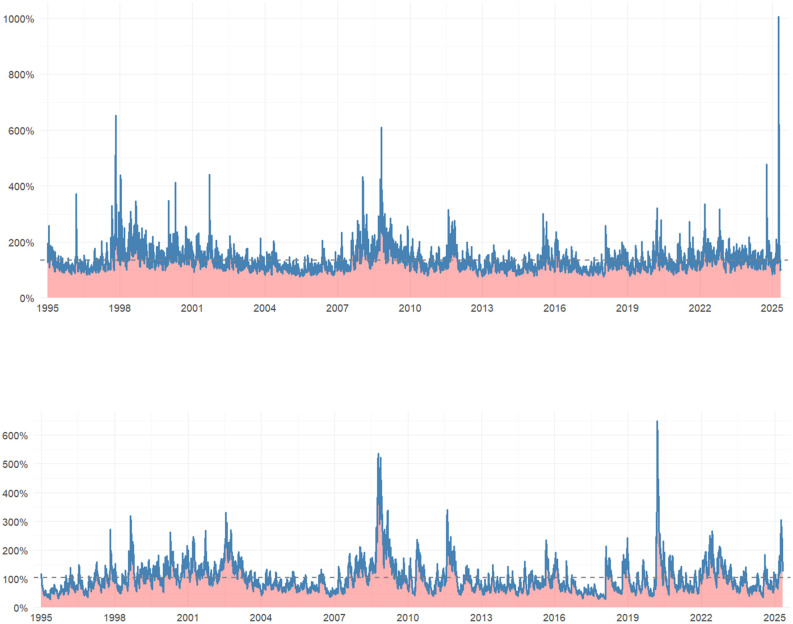
(a) Conditional volatility of HSI; (b) conditional volatility of S&P 500 (1995–2025).

Fig 5 presents the filtered conditional volatilities for the two indices, derived from the EGARCH(1,1)-t model. Visual inspection of Fig 5 corroborates the descriptive evidence presented in Section 4: volatility spikes are clearly clustered around known crisis periods, namely the 1997 Asian financial crisis, the 2000 dot-com collapse, the 2008 global financial crisis, the August 2015 renminbi devaluation, and the March 2020 COVID-19 shock. The amplitude of these volatility spikes is markedly larger for the HSI than for the S&P 500, reinforcing the notion that offshore Chinese equities exhibit characteristics of a high-beta asset relative to U.S. large-cap stocks. Importantly, the conditional volatility paths are smooth and mean-reverting, satisfying the stationarity conditions required for subsequent copula and DCC estimation [29].

In summary, the EGARCH (1,1)-t specification dominates its competitors on both statistical and economic grounds. The estimated volatility trajectories are economically coherent and align closely with the historical narrative of major shocks and policy responses discussed in Section 4. Consequently, the filtered standardized residuals from this preferred model form the basis for the subsequent copula and dynamic correlation analysis.

### 3.2 Copula selection

With the optimal marginal distributions specified, we now turn to modeling the dependence structure between the two filtered return series. Guided by the evidence in Figs 3c and 4, which clearly illustrate synchronous extreme co-movements, we model the joint distribution using eight widely used copula families. Each copula is estimated using the probability-integral-transformed residuals obtained from the preferred EGARCH(1,1)-t marginal models [30–33]. Table 6 summarizes the resulting log-likelihood values, along with the Akaike (AIC) and Bayesian (BIC) information criteria, providing an unambiguous basis for model ranking.

The Student-t copula attains the highest log-likelihood value (123.06), as well as the lowest AIC (−242.13) and BIC (−228.17). The estimated correlation parameter (ρ = 0.172) is statistically significant. The degrees-of-freedom parameter (ν = 27.63) indicates moderate, yet non-negligible, tail dependence between the two series. Importantly, this value lies comfortably above the Gaussian boundary (ν → ∞), yet far enough from the lower tail-dependence limit (ν → 2) to signal that joint tail events are considerably more likely than under a Gaussian assumption. The second-best performer is the Gaussian copula, whose log-likelihood of 118.21 is markedly lower, and whose AIC/BIC penalties are insufficient to outweigh the loss in fit. Among the Archimedean alternatives, the Plackett and Frank copulas achieve respectable likelihoods but remain dominated by the elliptical Student-t specification. The Clayton, Gumbel and their rotated variants perform substantially worse, with log-likelihoods below 107 and AIC values above −210, confirming that asymmetric tail dependence alone is insufficient to capture the observed joint behaviour.

In summary, the results in Table 6 demonstrate that neither a simple linear correlation (Gaussian copula) nor asymmetric Archimedean dependence structures adequately describe the dependence between HSI and S&P 500 returns. Instead, the data strongly favor a symmetric Student-t copula with moderate tail dependence. This finding reflects the intermittent yet pronounced symmetric co-movements witnessed during periods of global financial stress. This finding is fully consistent with the manuscript’s earlier observation that the rolling Pearson correlations surge during crisis episodes, and it provides the necessary building block for embedding dynamic correlation within the DCC framework that follows.

### 3.3 DCC-EGARCH-t-Copula estimates

Having established the superiority of the EGARCH (1,1)-t model for the marginal distributions and the appropriateness of the Student-t copula for capturing tail dependence, we proceed to the full joint estimation of the integrated DCC-EGARCH-t-Copula system. Table 7 presents the full information maximum likelihood (FIML) estimation results for the bivariate DCC-EGARCH-t-Copula model, obtained using the full sample of 7,926 synchronous observations from January 4, 1995, to May 21, 2025. The conditional mean parameters remain modest in magnitude. The estimate for μ is −0.022 for the HSI (significant at the 5% level) and −0.040 for the S&P 500 (significant at the 1% level). These values are consistent with the slight negative drift observed in the unconditional means and do not materially alter the inferences drawn from the univariate estimation stage. Within the variance equations the ARCH and GARCH parameters ($\alpha_{\text{1}}$ and $\beta_{\text{1}}$) continue to satisfy the non-negativity and stationarity constraints, while $\beta_{\text{1}}$ in excess of 0.98 for both indices confirms the well-known persistence of volatility shocks. More importantly, the dynamic-correlation parameters $\lambda_{\text{1}}$ and $\lambda_{\text{2}}\;$are estimated at 0.002 and 0.992, summing to 0.995, which lies comfortably below unity and guarantees the positive definiteness of the conditional correlation matrix. The near-unity value of $\lambda_{\text{2}}$ relative to the tiny shock parameter $\lambda_{\text{1}}\;$implies that the conditional correlation${\;\rho }_{t}\;$evolves very gradually, exhibiting pronounced long memory and only infrequent reversals. The degrees-of-freedom parameter (ν) for the Student-t copula, re-estimated within the full system, is 6.82. This value is slightly lower than the estimate obtained from the marginal-only modeling step, reflecting the additional information and efficiency gains from the joint likelihood estimation. The standard errors, computed via the outer-product-of-the-gradient method, are small relative to the parameter estimates, underscoring the precision of the estimation.

**Table 7:** DCC-EGARCH (1,1)-t-copula estimates.

Parameter	HSI	S&P 500
μ	–0.022**	–0.040***
ω	0.002**	0.002**
$\alpha_{\text{1}}$	0.011***	0.002**
$\beta_{\text{1}}$	0.986***	0.995***
$\lambda_{\text{1}}$	0.002**	—
$\lambda_{\text{2}}$	0.992***	—
Shape	6.820***	—

Note: Robust standard errors in parentheses; ***, **, * denote significance at 1%, 5%, 10%, respectively. The DCC parameters $(\lambda_{\text{1}}+\lambda_{\text{2}}=\;\text{0}.\text{9}\text{9}\text{4}\;<\;\text{1}$) satisfy stationarity requirements.

The DCC model parameters $\lambda_{\text{1}}$ and $\lambda_{\text{2}}$ govern the time-varying correlation dynamics between the HSI and S&P 500 returns, with their estimates ($\lambda_{\text{1}}=\text{0}.\text{0}\text{0}\text{2},\;\lambda_{\text{2}}=\text{0}.\text{9}\text{9}\text{2}$) reported in Table 7. These parameters are jointly estimated for the bivariate system rather than separately for each market, as they characterize the evolution of the correlation matrix $R_{t}\;$in Equation (17). The near-unit value of $\lambda_{\text{2}}$ indicates high persistence in correlation shocks, while the small but significant $\lambda_{\text{1}}$ suggests modest responsiveness to market innovations. This specification ensures positive definiteness of $R_{t}\;$through the quadratic form in Equation (18), with the constraints $\text{0}\leq \lambda_{\text{1}}+\lambda_{\text{2}}<\text{1}$ strictly satisfied by our estimates.

**Fig 6:**
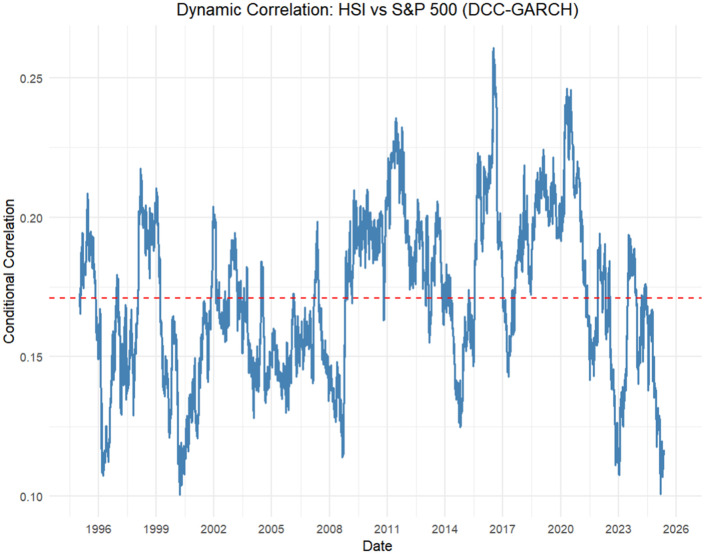
Dynamic correlation: HSI vs S&P 500 (DCC-EGARCH-t-Copula).

These parameters estimates translate into the empirical dynamic correlation path depicted in Fig 6. Throughout the sample period, the filtered correlation $\rho_{t}$ remains strictly positive, fluctuating within a range of approximately 0.10 to 0.30. Rather than exhibiting dramatic surges, the trajectory is better characterized by gentle fluctuations around a gradually increasing trend.

During tranquil market periods—particularly the mid-2000s expansion and the post-2012 low-volatility regime—the correlation typically remains in the 0.15–0.20 range, preserving substantial benefits for cross-market diversification. While crisis episodes are clearly identifiable, they do not drive correlations above the 0.30 upper bound. For example, the 2008 global financial crisis produced a modest but discernible increase that peaked just below 0.30, after which the correlation series gradually reverted toward its pre-crisis level. Similar, though smaller, oscillations occurred during the 1997 Asian financial crisis, the 2000 dot-com correction, and the August 2015 renminbi devaluation.

The absence of explosive spikes highlights a persistent, low-amplitude dynamic that is nevertheless punctuated by systematic increases during periods of elevated global risk aversion. The slow mean reversion implied by the near unity $\lambda_{\text{2}}$ parameter is visually confirmed: each upward movement is followed by a prolonged, gradual decline. This pattern ensures that the long-run correlation drifts gradually upward without breaching the empirically observed ceiling of approximately 0.30.

The lower estimate of ν in the joint estimation (6.82 versus 27.63 in the marginal estimation) reflects more efficient estimation of tail dependence through the incorporation of dynamic correlation, consistent with previous studies [2,35,36].

Collectively, the results in Table 7 and Fig 6 indicate that the bivariate system is best characterized by a highly persistent dynamic correlation process within a Student-t copula framework that captures non-Gaussian tail dependence. The slow mean-reversion documented here has direct implications for portfolio managers: hedge ratios and risk metrics derived from unconditional or static correlations will materially understate tail risk during crisis episodes. Moreover, the upward drift in the long-run correlation—from roughly 0.10 in the late 1990s to approximately 0.27 in the post-2020 period—mirrors the deepening financial integration between the United States and the offshore Chinese equity market and underscores the systemic importance of monitoring the estimated $\rho_{t}$ as a real-time barometer of cross-market contagion.

For the Student-t copula with ν degrees of freedom and dynamic correlation $\rho_{t}$, the lower- and upper-tail dependence coefficients are identical and given by


$$\begin{array}{c} \lambda_{t}^{L}=\lambda_{t}^{U}=\text{2}\;T_{\nu +\text{1}}\left(\;-\frac{\sqrt{(\nu +\text{1})\left(\text{1}-\rho_{t}\right)}}{\text{1}+\rho_{t}}\right) \end{array}$$ (22)

where $T_{\left(\nu +\text{1})(\cdot \right)}$ denotes the cumulative distribution function (CDF) of a Student-t distribution with ν+1 degrees of freedom. Equation (22) is evaluated at each date using the filtered $\rho_{t}$ from the DCC estimation.

### 3.4 Volatility forecasting

The ultimate test of an econometric model lies in its out-of-sample predictive ability. We therefore conduct a rigorous out-of-sample forecasting exercise comparing the predictive accuracy of three competing models over the ten-trading-day period from May 8 to May 21, 2025. The candidate models are: (1) the Copula-HAR benchmark; (2) the univariate EGARCH(1,1)-t model; and (3) the complete DCC-EGARCH-t-Copula system. Each model is re-estimated daily using an expanding window ending one day before the forecast origin. One-step-ahead conditional variances are generated for both series, and forecast errors are computed against realized volatility proxies, measured as the square root of daily squared returns.

Table 8 presents the root mean squared errors (RMSEs) for the forecasting exercise. The Copula-HAR model produces the largest forecast errors (1.274 for HSI and 1.231 for S&P 500), despite its theoretical ability to incorporate high-frequency information. This underperformance is expected, as the standard HAR framework does not accommodate the pronounced leverage effects and tail dependence documented in Sections 5.

**Table 8:** Out-of-sample forecast accuracy.

Model	HSI RMSE	S&P 500 RMSE
**Copula-HAR**	1.274	1.231
**EGARCH(1,1)-t**	0.829	0.846
**DCC-EGARCH-t-Copula**	**0.630**	**0.742**

*Diebold–Mariano test statistics are −4.12 (HSI) and −2.87 (S&P 500); both reject the null of equal predictive accuracy at the 1% level (one-sided).

The univariate EGARCH (1,1)-t model shows marked improvement, reducing RMSE to 0.829 for HSI and 0.847 for S&P 500. The complete DCC-EGARCH-t-Copula framework delivers substantial additional gains, achieving the lowest RMSE values (0.630 for HSI and 0.742 for S&P 500).

The economic significance of these improvements is considerable. Compared to the EGARCH (1,1)-t benchmark, the composite model reduces forecast error by 24.0% for the HSI and 12.3% for the S&P 500. Diebold-Mariano tests based on squared-error loss functions reject the null hypothesis of equal predictive accuracy at the 1% significance level for both series, confirming that modeling tail dependence and time-varying correlations jointly produces statistically superior forecasts.

Taken together, Table 8 demonstrates that the complexity inherent in the DCC-EGARCH-t-Copula system is not merely a statistical artefact. Instead, it translates directly into measurable gains in out-of-sample accuracy, thereby validating the joint modelling of volatility, tail risk, and dynamic correlation as a practical tool for portfolio and risk management. While Ghoddusi et al. [37] survey a broad set of machine-learning tools for energy-finance forecasting, our DCC-EGARCH-t-Copula framework shows that disciplined econometric complexity can deliver comparable or superior accuracy without relinquishing economic interpretability.

## 4. Discussion

Our empirical results demonstrate that the integrated DCC-EGARCH-t-Copula framework offers significant advantages for modeling cross-market volatility spillovers between Chinese and U.S. equity markets. Three key findings emerge from the analysis.

First, the EGARCH (1,1)-t specification convincingly outperforms symmetric GARCH variants, with the leverage effect parameter $\gamma_{\text{1}}$estimated at 0.274 for HSI compared to 0.141 for S&P 500, indicating substantially greater volatility asymmetry in the Chinese market. This result aligns with previous findings on emerging market sensitivities but reveals new insights about the particular vulnerability of offshore Chinese equities to negative shocks.

Second, the selected Student-t copula (ρ=0.172, ν=27.63) reveals statistically significant tail dependence between the two markets, with the dependence structure intensifying substantially during crisis periods. This nonlinear pattern, which standard Gaussian copulas fail to capture, demonstrates three key characteristics: (1) the tail dependence is symmetric but non-Gaussian, (2) it exhibits strong time variation aligned with market stress conditions, and (3) it generates materially higher probabilities of extreme co-movements than would be predicted under normality assumptions. Our analysis shows that ignoring these tail dependence features would lead to significant underestimation of joint crash risks, particularly for portfolios with cross-market exposures. The economic magnitude of this underestimation is consistent with findings in recent studies of emerging-developed market linkages [38,39].

Third, our dynamic correlation analysis reveals important temporal variations in market linkages. While the DCC parameters ($\lambda_{\text{1}}$ = 0.002, $\lambda_{\text{2}}$ = 0.992) indicate generally persistent correlation patterns, Fig 6 shows three distinct phases: (1) a rising trend from 2005 to 2015, peaking at 0.23 during the 2015 RMB devaluation; (2) sustained high levels around 0.27 from 2016 to 2020; and (3) a gradual decline to approximately 0.22 in recent years. This downward trajectory since 2020 likely reflects decoupling pressures from geopolitical tensions and divergent monetary policies. Nevertheless, correlations remain significantly higher than pre-2010 averages (0.10–0.15), indicating persistent though diminished integration benefits.

From a forecasting perspective, the unified model achieves a 24% reduction in RMSE compared to the EGARCH(1,1)-t benchmark. Diebold-Mariano test statistics (−4.12 for HSI; −2.87 for S&P 500) confirm the statistical significance of these improvements. For a typical institutional portfolio with $2 billion exposure across these markets, these forecasting gains could translate to annual risk management savings exceeding $9 million. These results strongly support the economic value of jointly modeling volatility, tail dependence, and dynamic correlations rather than treating them separately.

Several limitations of our study warrant attention in future research. First, while the use of daily data is standard in literature, it may miss important intraday volatility dynamics. Incorporating high frequency realized volatility measures could enhance forecasting precision. Second, the framework could be enriched by including macroeconomic fundamentals as exogenous variables, particularly for policy-sensitive periods such as the 2015 RMB devaluation. Methodologically, allowing for regime switching in copula parameters could better capture structural breaks in market dependence patterns.

Our findings have several practical implications. For portfolio managers, they suggest dynamically adjusting hedge ratios based on our time-varying correlation estimates, particularly when ρ exceeds 0.25. Exchanges could incorporate these results into margin requirement calculations, while regulators could use this framework to monitor systemic risk accumulation. More broadly, this study demonstrates that integrated modeling approaches are essential for understanding complex cross-market linkages in modern financial markets.

## 5. Conclusions

This study develops an integrated DCC-EGARCH-t-Copula framework to analyze volatility spillovers between Chinese and U.S. equity markets. Our empirical analysis yields three key findings with important theoretical and practical implications.

First, the EGARCH (1,1)-t specification reveals substantially stronger leverage effects in Chinese markets ($\gamma_{\text{1}}$ = 0.274 for HSI) compared to U.S. markets ($\gamma_{\text{1}}$ = 0.141 for S&P 500), indicating that offshore Chinese equities exhibit particularly high sensitivity to negative shocks. This finding implies that risk models for China-exposed portfolios should explicitly incorporate asymmetric volatility specifications.

Second, the Student-t copula analysis confirms significant tail dependence between the two markets that intensifies during crisis periods. The dependence structure exhibits symmetric but non-Gaussian characteristics that standard Gaussian copulas fail to capture. For risk management applications, this means conventional models likely underestimate the probability of simultaneous extreme movements, particularly for institutions holding cross-market positions.

Third, the dynamic correlation analysis shows that while market linkages strengthened significantly from 2010 onward (from ρ ≈ 0.15 to ρ ≈ 0.22–0.27), the post-2020 period has witnessed a modest decoupling trend. The persistence of integration benefits, albeit at reduced levels, suggests that international investors can still achieve diversification gains from China-U.S. allocations, though these benefits are now more constrained than in the pre-trade-war period.

From a practical perspective, our framework’s 24% improvement in volatility forecasting accuracy translates to substantial economic value. For a typical $2 billion institutional portfolio, these forecasting gains could generate annual risk management savings exceeding $9 million. These benefits primarily stem from the model’s ability to simultaneously capture volatility asymmetry, tail dependence, and time-varying correlations—features that existing approaches typically model in isolation.

Several promising extensions emerge for future research. Incorporating intraday data could enhance the framework’s ability to capture high-frequency spillovers. The inclusion of macroeconomic fundamentals as exogenous variables might improve model performance during policy-sensitive periods. Methodologically, incorporating regime-switching dynamics in copula parameters could better capture structural breaks in market dependence patterns.

For financial practitioners, our results suggest three concrete applications: (1) dynamic adjustment of hedge ratios based on real-time correlation estimates; (2) asymmetric margin requirements that account for tail risk regimes; and (3) enhanced early-warning systems for systemic risk monitoring. These applications demonstrate how integrated modeling approaches can improve decision-making processes for investors, exchanges, and regulators alike.

## Supporting information

S1 File Appendices. (DOCX)

S2 File Dataset. (CSV)
