# Supplementary material for: Cross-market volatility spillovers between China and the United States: A DCC-EGARCH-t-Copula framework with out-of-sample forecasting
Source: PLoS One. 2025 Oct 17;20(10):e0333794. doi: 10.1371/journal.pone.0333794 (PMC12533925; doi:10.1371/journal.pone.0333794)
Supplement: S1 File — (DOCX) [file pone.0333794.s001.docx]

Appendix A

**Multi-Step Volatility Forecast for EGARCH (1,1)-t**

Let${RV}_{t}$denote the realized volatility proxy, calculated as the square root of daily squared returns [1]. All multi-step forecasts are derived from the EGARCH (1,1)-t structure in Equation (A1). The conditional log-variance follows

$$\begin{aligned} \ln\sigma_{t}^{2}=\omega+\alpha\left( \left| \varepsilon_{t-1} \right|-E\left| \varepsilon_{t-1} \right| \right)+\gamma\varepsilon_{t-1}+\beta\ln\sigma_{t-1}^{2}\#\left( A1 \right) \end{aligned}$$

where $\varepsilon_{t}=\sigma_{t}z_{t}$ and$z_{t}\sim t_{\nu}\left( 0,1 \right)$.

Closed-form forecast of the log-variance k-steps ahead is obtained by iterating expectations:

$$\begin{aligned} E_{t}\left[ \ln\sigma_{t+k}^{2} \right]= \omega\sum_{j=0}^{k-1}\beta^{j}+\beta^{k}\ln\sigma_{t}^{2}+\left( \alpha E\left| z \right|+\gamma Ez \right)\sum_{j=1}^{k}\beta^{j-1}\#\left( A2 \right) \end{aligned}$$

For the Student-t with $\nu>2$,

$$\begin{aligned} E\left| z \right|=\frac{2\sqrt{\nu}\Gamma\left( \frac{\nu+1}{2} \right)}{\sqrt{\pi}\left( \nu-1 \right)\Gamma\left( \frac{\nu}{2} \right)},\quad\quad Ez=0\#\left( A3 \right) \end{aligned}$$

Following the log-normal approximation approach of Bollerslev et al. [2], the conditional variance in levels is obtained by exponentiating the log-variance expectation and adjusting for convexity:

$$\begin{aligned} \sigma_{\left( t+k | t \right)}^{2}=\exp\left\{ E_{t}\left[ \ln\sigma_{t+k}^{2} \right]+½ Var_{t}\left[ \ln\sigma_{t+k}^{2} \right] \right\}\#\left( A4 \right) \end{aligned}$$

Where

$$\begin{aligned} Var_{t}\left[ \ln\sigma_{t+k}^{2} \right]=\frac{\alpha^{2}\left( 1-\beta^{2k} \right)}{1-\beta^{2}}Var\left( \left| z \right| \right)\#\left( A5 \right) \end{aligned}$$

The variance term$Var_{t}\left[ \ln\sigma_{t+k}^{2} \right]$ arises from the accumulation of shocks in the log-variance process (see Appendix B of Patton [3] for derivation). And

$$\begin{aligned} Var\left( \left| z \right| \right)=1-\left( E\left| z \right| \right)^{2}\#\left( A6 \right) \end{aligned}$$

Plugging (A2)–(A6) into (A4) yields a fully analytic $k$-day-ahead volatility forecast.

Appendix B

**Loss Function Decomposition for Copula-HAR**

The Copula-HAR log-likelihood combines:

(i) The copula density$c_{har}(\cdot)$ with time-varying dependence parameters

(ii) The marginal density of realized volatility$f_{RV}(\cdot)$

Following the framework of Engle and Patton [4]. Let $\omega_{t}= \left( RV_{t}, RV_{\left\{ t-1 \right\}}, RV_{\left\{ t-5 \right\}} \right)^{'}$be the HAR regressors and $u_{t}$ the probability integral transform of residuals. The Copula-HAR log-likelihood is

$$\begin{aligned} \mathcal{L=}\sum_{t=1}^{T} \left[ \ln c_{\text{har}}\left( u_{t};\theta\right)+\ln f_{\text{RV}}\left( RV_{t} \mid\omega_{t} \right) \right]\#\left( B1 \right) \end{aligned}$$

Where$c_{har\left( \cdot\right)}$is the time-varying copula density. The first-order condition $\frac{\partial\mathcal{L}}{\partial\theta}= 0$ yields the score vector

$$\begin{aligned} s_{t}=\frac{\partial\ln c_{\text{har}}}{\partial\theta}+\frac{\partial\ln f_{\text{RV}}}{\partial\theta}\#\left( B2 \right) \end{aligned}$$

And the sandwich covariance estimator $\hat{\Omega}= \left( \hat{H} \right)^{\left\{ -1 \right\}}\hat{S}\left( \hat{H} \right)^{\left\{ -1 \right\}}$ is used to obtain robust standard errors. The score vector$s_{t}$accounts for parameter uncertainty from both the copula and marginal estimation stages, with the information matrix equality ensuring consistent standard errors [5].

References

1. Andersen T. G., Bollerslev T., Diebold F. X., and Labys P. Modeling and forecasting realized volatility. *Econometrica*. 2003;71(2): p. 579–625. doi: <https://doi.org/10.1111/1468-0262.00418>
2. Bollerslev T., Patton A. J., and Quaedvlieg R. Exploiting the errors: a simple approach for improved volatility forecasting. *J Econometrics.* 2016;192(1): p. 1–18. doi: <https://doi.org/10.1016/j.jeconom.2015.10.007>
3. Patton A. J. Copula methods for forecasting multivariate time series. In: Elliott G, Timmermann A, editors. *Handbook of economic forecasting*. Vol. 2. Amsterdam: Elsevier; 2013. p. 899–960. doi: <https://doi.org/10.1016/B978-0-444-62731-5.00016-6>
4. Engle R. F., and Patton A. J. What good is a volatility model? *Quant Financ*. 2001;1(2): p. 237–245. doi: <https://doi.org/10.1088/1469-7688/1/2/305>
5. White H. Maximum likelihood estimation of misspecified models. *Econometrica.* 1982;50(1): p. 1–25. doi: <https://doi.org/10.2307/1912526>
